# A fossil *Diploglossus* (Squamata, Anguidae) lizard from Basse-Terre and Grande-Terre Islands (Guadeloupe, French West Indies)

**DOI:** 10.1038/srep28475

**Published:** 2016-06-29

**Authors:** Corentin Bochaton, Renaud Boistel, Fabrice Casagrande, Sandrine Grouard, Salvador Bailon

**Affiliations:** 1 Laboratoire “Archéozoologie et Archéobotanique: Sociétés, Pratiques et Environnements” – UMR 7209 – CNRS, MNHN - Muséum national d’Histoire naturelle - Sorbonne Universités, 55 rue Buffon, CP 56, 75005 Paris, France; 2 Institut de Systématique, Évolution, Biodiversité – UMR 7205 – CNRS, MNHN, UPMC, EPHE- Muséum national d’Histoire naturelle - Sorbonne Universités, 57 rue Cuvier, CP 30, 75005, Paris, France; 3 Institut de Paléoprimatologie et de Paléontologie Humaine – UMR 7262 – CNRS, Université de Poitiers, UFR SFA - Bât. B35 - 06 rue Michel Brunet - TSA 51106, F-86073 Poitiers, France; 4 Institut National de Recherche en Archéologie préventive (INRAP) Route de Dolé, Maison Lacascade 97113, Gourbeyre, Guadeloupe; 5 Laboratoire “Histoire naturelle de l’Homme préhistorique » – UMR 7194– CNRS, MNHN - Muséum national d’Histoire naturelle - Sorbonne Universités, 75013 Paris, France

## Abstract

Today, Diploglossine lizards (Anguidae) are common on the Greater Antillean Islands (West Indies), where they are represented by many endemic species. However these lizards are very rare on the Lesser Antillean Islands, where they are only represented by a single species, the Montserrat galliwasp (*Diploglossus montisserrati*). Here, we show that diploglossine lizards were present in the past on other Lesser Antillean islands, by reporting the discovery of Anguidae fossil remains in two Amerindian archaeological deposits and in a modern deposit. These remains are compared to skeletons of extant diploglossine lizards, including *D. montisserrati*, using X-ray microtomography of the type specimen of this critically endangered lizard. We also conducted a histological study of the osteoderms in order to estimate the putative age of the specimen. Our results show that the fossil specimens correspond to a member of the *Diploglossus* genus presenting strong similarities, but also minor morphological differences with *D. montisserrati*, although we postulate that these differences are not sufficient to warrant the description of a new species. These specimens, identified as *Diploglossus* sp., provide a new comparison point for the study of fossil diploglossine lizards and reflect the historical 17^th^ century mentions of anguid lizards, which had not been observed since.

Two genera of diploglossine lizards currently occur in the West Indies, the *Celestus* Gray, 1839 and *Diploglossus* Wiegmann, 1834 genera. Both are present in the Greater Antilles, the large northern West Indian islands, where they are represented by 25 endemic species (21 *Celestus* and 4 *Diploglossus*)^1^. However, a single diploglossine species occurs in the Lesser Antilles, the smaller southern West Indian Islands. This species, the Montserrat galliwasp, *Diploglossus montisserrati* Underwood, 1948, is restricted to Montserrat Island and is endemic to this island. As regards the colonization scenario of the West Indian Islands, Hass *et al*.^2^ suggested that *Diploglossus* first colonized the Greater Antilles more than 20 My ago, and that *Celestus* arrived during a second phase around 10 My ago. Strahm and Schwartz^3^ postulated that *Celestus* colonized the Greater Antilles from Central America and that *Diploglossus* arrived from South America and the Lesser Antilles, on the basis of the modern distribution of the genera and the occurrence of *D. montisserrati* on Montserrat Island. However, the morphological similarity established between *D. montisserrati* and the mainland *D. monotropis* and the possible morphological differences observed with the Greater Antillean taxa by Underwood^4^ could indicate a recent colonization of the Lesser Antilles from South America independently of the Greater Antilles colonization. Yet until now, the lack of molecular data concerning the Montserrat galliwasp and the difficulty involved in clearly delimiting the *Celestus* and *Diploglossus* genera^5^,^6^ complicated the establishment of a coherent colonization scenario. Yet, these biogeographic problems raise the question of the possible past occurrence of now extinct diploglossine lizards elsewhere on the Lesser Antillean Islands, other than Montserrat, and consequently, the question of the chronology and the reasons for the extinction of these lizards.

This study attempts to tackle these questions by presenting remains attributed to a previously unknown fossil *Diploglossus* lizard found among the archaeological material (pottery, stone, bone and shell tools, remains of consumed animals) from three archaeological and natural sites in Guadeloupe. The first site, which provided nearly all the studied remains is called “Saint-Rose la Ramée”, and is situated in the north of Basse-Terre Island, the largest island of the Guadeloupe Archipelago, near the city of Saint-Rose (Fig. 1). It was excavated in 2007 by F. Casagrande (Institut National de Recherche en Archéologie Préventive –INRAP) during a rescue excavation. This Amerindian settlement is a pre-Columbian village occupied between 640 and 950/1000 AD. The second archaeological site, “Cathédrale de Basse-Terre”, was excavated in the context of a recue excavation in 2002 by D. Bonnissent (INRAP). It yielded a single anguid bone (a trunk vertebra) recovered from a Pre-Columbian layer dated from the early Saladoid period on the basis of archaeological pottery^7^. This site is also situated on Basse-Terre Island but is located in the south of the island (Fig. 1). The third site, “la Grotte des Bambous” also only provided one of the studied bones but is situated on Grande-Terre Island, the second largest island of the archipelago, near the “Patate” ravine (Fig. 1). This latter site is a natural deposit excavated by A. Lenoble in 2014^8^ and dates from the colonial period but precise chronological data are not yet available.

This discovery is important since very little is known about the history of anguid lizards in the Lesser Antilles and the only comparative specimen is the Montserrat galliwasp from the eponymous island. Thus, in addition to a detailed morphological analysis of the fossil specimens, we also compare it to the Montserrat galliwasp using X-ray microtomography of the type specimen of this IUCN listed as a critically endangered lizard^9^.

## Material and Methods

A 2 mm mesh screen was used to collect osteological remains from the archaeological sediments during the excavations. Among the small faunal remains, 176 (26 bones and 150 osteoderms) were attributed to an anguid lizard.

The 174 remains collected from the Saint-Rose la Ramée site come from four different stratigraphic units (US 1956; US 2043; US 2058 and US 1530), located in four different loci of the site. Three of these units were radiocarbon dated at the Beta Analytic Radiocarbon Dating Laboratory (Miami, U.S.A.): 1090 +/− 40 BP (Cal. 880 to 1020 AD) for US 1956 and 2043 (ref. Beta 244995 and Beta 244996), and 1250 +/− 40 BP (Cal. 670 to 880 AD) for US 1530 (ref. Beta 244991)^10^. The sole element (a trunk vertebra) from the Cathédrale de Basse-Terre site was located in US 1086, containing Cedrosan-Saladoid pottery and thus probably dating from between 300 and 900 AD^7^. The only bone (a dentary) from the Grotte des Bambous site was located a layer dated from modern period but lacking precise chronological attribution^8^.

Taxonomic identification was based on characters described in the literature and comparative diploglossine lizard osteological specimens from the Museum of Comparative Zoology (Harvard, USA): *Diploglossus millepunctatus* (MCZ R-130071), *Diploglossus monotropis* (MCZ R-22912), *Diploglossus pleii* (MCZ R-131518), *Celestus costatus* (MCZ R-65006), *Celestus hewardii* (MCZ R-45170), and *Ophiodes striatus* (MCZ R-7271 and MCZ R-20669). An osteological comparison with *D. montisserrati* was undertaken using X-ray microtomography (voxel size = 84.2–62.9 μm) of the type specimen (MCZ R-76924), previously used by Bochaton *et al*.^11^. Direct volume rendering was used to visualize the subset of selected voxels of the skeleton in AVIZO v. 7.1 and 6.1 (VSG, SAS, Merignac, France, http://www.vsg3d.com), after applying IMAGEJ to mask the anatomical structures of no interest here. In addition to the examination of the external morphology, two fossil osteoderms corresponding to the two different morphotypes highlighted by our morphological observations were prepared in order to observe their histological organization. They were embedded in polyester resin, sectioned along their transverse axes, mounted on a glass slide, and polished to obtain thin sections with a thickness of about 100 μm. The obtained slides were then observed with a compound microscope with natural and polarized light. Measurements were taken on bones using a digital dial caliper [IP 67 (Mitutoyo Corporation, Japan)] and using the AVIZO 6.1 3D measurement tool on the X-ray microtomography.

## Systematic Paleontology

REPTILIA Laurenti, 1768, SQUAMATA Oppel, 1811, ANGUIDAE Gray, 1825, *DIPLOGLOSSUS* Wiegmann, 1834.

### Description

#### Material

One right maxilla fragment, one left quadrate bone, one left and one right dentary, eleven trunk vertebrae, two sacral vertebrae, nine caudal vertebrae and 150 osteoderms.

#### Maxilla

The maxilla (Fig. 2A) is documented by a single fragment corresponding to the posterior half of the bone. In lateral view, the fragment presents three labial foramina. The facial process is posterodorsally elongated, with a posteriorly inclined and slightly concave dorsal margin, and a stepped posterior part. The morphology of the anterior half of this broken facial process is unknown. A small over-elevated area presenting dermal ornamentation formed of small pits and short furrows can be observed near the anterior extremity of the fragment, above the first labial foramen. Directly above the dental crest, the whole length of the bone is perforated by many small foramina. The zygomatic process is short and posteriorly bifurcated with a pointed posterodorsal process and a shorter posteroventral process in a more medial position. These two processes are separated by a deep semi-oval notch which received the anteromedial branch of the ectopterygoid in the living animal. In dorsal view, a moderately developed and slightly incurved supradental shelf (*sensu* Rage and Augé)^12^ is visible along the whole length of the dental row. A well-developed oblique ridge (carina maxillaris *sensu* Müller)^13^ crosses half of the supradental shelf and links the internal face of the facial process to the dorsal surface of the supradental shelf near the anterior extremity of the fragment. The posteroventral margin of this ridge delimits a deep maxillary fossa. The palatal process is medially well developed, but the medial margin is broken. This process presents a rugose surface corresponding to the zone of contact with the palatine bone. The very posterior part of the supradental shelf presents a wide medial notch, anteriorly delimited by a medial spine for the joint with the anteromedial branch of the ectopterygoid in the living specimen. The anterior half of the supradental table (*sensu* Rage and Augé)^12^ is perforated by two infraorbital foramina. The posterior part of the bone is divided into two portions, an elongated and pointed lateral portion (posterodorsal process described above) bearing a longitudinal dorsal jugal groove, and a shorter and blunt ventromedial portion (posteroventral process described above). A small foramen occurs at the base of the separation of these two portions. In medial view, the supradental shelf is dorsally slightly convex. The fragment presents seven dental positions bearing pleurodont and heterodont teeth with striated apices. The first two unbroken teeth are straight and unicuspid with lateromedially compressed apices forming a longitudinal cutting edge in ventral view. The next four teeth become abruptly larger and bulbous with a rounded apex (amblyodont teeth) and a very reduced cutting edge. The striations observed all around the crown of the teeth are formed by many parallel dorsoventrally oriented striations, which converge towards the cutting edge.

#### Quadrate

The only collected quadrate (Fig. 2C) is whole with a maximal height of 10 mm. It is rectangular in anterior and posterior views and is triangular-shaped with a ventral apex in medial and lateral views. In posterior view, the bone presents a deep tympanic fossa bordered by a well-developed tympanic crest with a straight lateral margin. The bone also bears a weakly developed pterygoid crest. This crest presents short and pointed medial expansion corresponding to the impression of the posterior process of the pterygoid. A small fossa also corresponding to the impression of the end of the posterior process of the pterygoid is present at the base of the posterior crest. In lateral view, the bone presents well-marked anterodorsal expansion of the tympanic crest (=columnar epiphysis *sensu* Fejérvary-Langh)^14^. In dorsal view, a tubular bony bridge links the cephalic condyle to the columnar epiphysis and closes the notch for the insertion of the squamosal. In ventral view, the mandibular condyle is wide and saddle-shaped.

#### Dentary

The most complete dentary discovered in the site of Saint-Rose la Ramée (Fig. 3B) bears 19 dental positions, with a 24 mm long dental row and a maximal length of 29.2 mm, and is broken in its posteroventral part. The second dentary discovered at the site of Grotte des Bambous does not seem to present any morphological differences with the first but is very broken and bears marked digestion traces. In lateral view, the bone has a slightly convex ventral margin and lacks dermal ornamentation. Five labial foramina are present on the anterior half of the bone at half its height. It bears two posterior processes of similar length: a posterodorsally oriented coronoid process and a posteriorly oriented supra-angular process. These processes are clearly separated by a deep coronoid incision and a deep and very rugged imprint of the anterolateral branch of the coronoid, which extends to the level of the last dental position and indicates an overlap of the coronoid on the dentary in the living animal. The occurrence and morphology of an angular process cannot be assessed because the posteroventral part of the bone is broken. The imprint of the anteromedial branch of the coronoid in medial view reaches the last dental position. The subdental shelf (*sensu* Rage and Augé)^12^ is ventromedially sloped, lacks *sulcus dentalis* (*sensu* Rage and Augé)^12^, except on the anterior extremity, and bears a notch corresponding to the anterodorsal margin of the anterior alveolar foramen under the 15–16^th^ dental positions. Although the subdental shelf is slightly worn, it is likely to lack a splenial spine. The posterior portion of the intramandibular septum is not ventrally fused with the dentary. The septum was prolonged by a surangular spine (*sensu* Klembara *et al*.)^15^ of unknown length. In medial and ventral views, the Meckelian’s groove is fully open, in a medial direction in the central and posterior parts and in a ventral direction in the anterior part. In ventral view, the imprint of the splenial bone is visible and indicates that the anterior quarter of the Meckelian groove (to the level of the 10^th^ dental position) was left uncovered by the splenial. The mandibular symphysis is small. The dentition is composed of pleurodont and heterodont teeth, which all bear a striated apex similar to those previously described on the maxilla. The eleven more anterior dental positions bear cylindrical teeth with a lateromedially flattened apex forming a dorsal longitudinal cutting edge. In the next four positions the teeth become wider and the apex is more rounded, but the longitudinal cutting edge is still present at the top of the apex. However, in the next three positions the teeth become even larger and bulbous (amblyodont teeth) and the cutting edge almost disappears. The size of the posterior teeth is reduced.

#### Trunk vertebrae

The 11 preserved trunk vertebrae (Fig. 4A) are procelous and possess a centrum length ranging from 3 mm to 5.5 mm. In dorsal view, each vertebra is longer than wide, presents well-marked medial constriction and well-expanded pre and post-zygapophysis. In ventral view, the centrum is triangular with well-defined lateral margins, and a wide and flattened hemal keel presenting posteriorly divergent lateral margins. Slight precondylar constriction occurs between the centrum and the condyle. In lateral view, the neural spine is longer than high and posteriorly sloped. The neural spine occupies the whole length of the neural arch and is posteriorly extended beyond the posterior limit of the post-zygapophysis. The vertebrae bear no zygosphene/zygantrum articulation. In anterior or posterior views, the pre-zygapophysis and post-zygapophysis are lateromedially sloped and form a right angle with the neural arch. The condyle and cotyle are dorsoventrally flattened.

#### Sacral vertebrae

The sacrum (Fig. 4B) is composed of the fusion of the two sacral vertebrae. The total length of the two fused centra is 7.1 mm and the maximal width containing the transverse process is 18.5 mm. In dorsal view, the neural arches are shorter than on the trunk vertebrae. Centra are nearly flat and fused together. The sacral transverse processes are long and lateroventrally oriented, those of the first sacral vertebra are twice as wide as those of the second sacral vertebra. The sacral processes of both of these vertebrae are very close to each other and are fused together in their distal half. In ventral view, centra are concave with a notch on the ventral margin of the cotyle of the first vertebra and two deep subcentral depressions on the second vertebra. In lateral view, the transverse processes formed a rounded articular surface with a slight posterior notch in contact with the ilium.

#### Caudal vertebrae

Nine elongated procelous caudal vertebrae (Fig. 4C) are preserved. In dorsal view, they bear anterolaterally oriented single transverse processes on the anterior third of their neural arch. A fracture plane cuts the transverse processes into two parts of unequal sizes; a very small anterior part corresponding to small basal-anterior tips of the process and a larger posterior part visible on all the vertebrae. In ventral view, a medial furrow occurs along the whole length of the centrum. A long posteroventrally oriented hemapophysis (“chevron” bones *sensu* Hoffstetter and Gasc)^16^, fused with the centrum, occurs in the posterior half of the centrum.

#### First osteoderm morphotype

The preserved osteoderms can be divided into two basic morphotypes. All of the osteoderms are non-compound; they are single, unfused, elements. The first morphotype is rounded and flat, and generally smaller than the second. Maximal osteoderm length is between 3.5 mm and 6 mm. They are thin and not laterally beveled, with a thickness of less than 12%, and mostly around 7% of their maximal length. In superficial view, they present a crescentic gliding surface (*sensu* Hoffstetter)^17^, corresponding to the overlap area with the osteoderm of the previous row. This gliding surface presents a posterior projection and a “cloud-like” radix system (*sensu* Strahm and Schwartz)^3^. The posterior portion is ornamented by small pits and ridges. The histological cross-section of one of these osteoderms (Fig. 5C) shows the occurrence of the three cortices previously described by Bochaton *et al*.^18^ in diploglossine lizard osteoderms. The superficial cortex is the thinnest and is composed of an intermediate parallel-fibered and lamellar bone tissue type. This layer presents evidence of superficial resorption since it is partly eroded in our specimen and also presents tenuous alternation of light and dark strata in polarized light. Such marks are more clearly visible in the basal layer. The central layer is the thickest and is composed of woven-fibered bone tissue with evidence of strong remodeling, as shown by large resorption bays. The basal layer is composed of parallel-fibered bone and presents around 10 alternations of thin dark and thicker light strata in polarized light (Fig. 5E). These alternations are interpreted as cyclical growth marks by Bochaton *et al*.^18^, and in this case they indicate that the specimens are around ten years old.

#### Second osteoderm morphotype

These osteoderms (Fig. 5B) are also simple osteoderms composed of a single element. However they are bigger than the previously described morphotype with a maximal length of 4 to 8 mm. They are thicker than the first morphotype with a thickness superior to 19%, and mostly around 25% of their maximal length, and they are quite strongly transversely arched rather than flat. In superficial view, their general shape is also different; most of them are rounded but some tend to be subrectangular. A gliding surface occupies the anterior third of the osteoderms, and general shape varies from sub-rectangular to crescent-shaped. There is no cloud-like radix or posterior projection. Unlike the first morphotype, the lateral tips of the osteoderms are laterally beveled and the posterior area presents ornamentation consisting of small pits and tubercles. The histological cross section of one of these thick elements (Fig. 5D) displays the three cortices previously described in the first morphotype, but also an additional cortex, very rich in transversely oriented Sharpey’s fiber, in the lateral margins of the sections (Fig. 5F). Finally, this osteoderm differs from the first morphotype by the higher number of alternations of thin dark and thicker light strata occurring in the basal layer (around 36) (Fig. 5G). All the specific characters observed on these osteoderms clearly indicate regenerated tail osteoderms, as previously described by Bochaton *et al*.^18^. Consequently, the number of cyclical deposits observed in the basal layer does not follow the same cyclicality as the non-regenerated osteoderms and thus cannot be used to estimate the age of a single individual.

#### Comments

All these elements were associated on the basis of their morphological affinities. Given the diverse origins of these remains from different sites and layers, they must represent at least six individuals. The measurements taken on the remains from Saint-Rose la Ramée, with the exception of a few small vertebrae, are slightly higher than those recorded on the X-ray microtomography of the *D. montisserrati* specimen with a snout-vent length (SVL) of 180 mm. Thus, the size of the fossil specimens from this site should be around 200 mm SVL. The occurrence of regenerated osteoderms shows that specimens from Saint-Rose la Ramée lost their tails during their lifetime. These remains, mostly found in archaeological deposits, do not present any traces of predation or anthropic actions.

### Identification

These fossil remains present several characters often associated with anguid lizards: (1) an incurved supradental shelf of the maxilla; (2) a free posteroventral margin of the intramandibular septum; (3) a fully open Meckelian groove partially filled by the splenial; (4) a notch for the anterior alveolar foramen in the subdental shelf of the dentary; (5) an anterolateral process on the coronoid; (6) teeth with striated apex; (7) a notch for the insertion of the squamosal bone on the quadrate; (8) hemapophysis fused with the centra of the caudal vertebrae; (9) osteoderms composed of a single element^19^,^20^,^21^,^22^,^23^,^17^,^24^. However the examination of the literature and osteological material shows that most of these characters are not clear autapomorphic Anguidae traits: although the occurrence of a free posteroventral margin of the intramandibular septum on the dentary (2) is described by several authors^19^,^20^,^23^, it seems to be absent in the *Pseudopus apodus* anguid^15^. The contribution of the dentary to the dorsal and anterior margin of the anterior alveolar foramen, which is materialized by a notch on the subdental shelf of the dentary (4), is also described as an anguid character^19^,^20^,^22^, but our observations show that this character is variable among Diploglossinae. Indeed the anterior alveolar foramen is sometimes fully placed on the splenial bone for the three genera observed here (*Diploglossus*, *Celestus* and *Ophiodes*). The occurrence of a fused hemapophysis on the caudal vertebrae (7) was listed as an Anguidae character by Conrad^20^, but is absent in *Diploglossus* according to Meszoely^25^. However, we recorded this character in all the observed diploglossine lizard skeletons. Lastly, the occurrence of teeth with a striated apex (5) was only observed in Diploglossinae by Meszoely^25^, but was reported by Klembara *et al*.^15^ in *Ophisaurus* and *Pseudopus*. These data show that most of these characters are probably subject to intraindividual and intra and interspecific variability among Anguidae, although their combined occurrence on our fossil does not cast any doubt on its identification as an Anguidae.

The fossil also presents characters currently recognized as traits of Diploglossinae: (1) rounded flat osteoderms, non-beveled along their lateral edges with a crescent-shaped gliding surface and a posterior projection^18^,^17^, (2) skink-type caudal autotomic vertebrae with a single transverse process divided by a fracture plane into a small anterior and larger posterior parts (Etheridge^26^ and pers. obs.), whereas other anguids present autotomic caudal vertebrae bearing two pairs of converging lateral processes, (3) centra of presacral vertebrae are triangular in ventral view, not flattened but with a slightly raised central portion (hemal keel) and with a slight precondylar constriction^25^, and (4) occurrence of a columnar epiphysis on the quadrate bone^27^. However, we found the hemal keel on presacral vertebrae (3) and the occurrence of a columnar epiphysis on the quadrate bone (4) to be absent in *Ophiodes*.

Additionally, among the three diploglossine lizard genera (*Diploglossus*, *Celestus* and *Ophiodes*), observations made on our comparative specimens show that the following characters differentiate the fossil from *Ophiodes*: (1) a tympanic and pterygoid crest and a columnar epiphysis on the quadrate bone (see also Alves *et al*.^28^), (2) a well-developed articular surface on the transverse process of the sacral vertebrae, (3) a hemal keel on trunk vertebrae, and (4) larger overall size of the elements than in extant *Ophiodes* species. Considering the two remaining diploglossine lizard genera (*Diploglossus* and *Celestus*) which occur in the West Indies, the fossil presents a series of characters present in *Diploglossus* and absent in *Celestus*: (1) a cloud-like radix system on the gliding surface of all the non-regenerated osteoderms (first morphotype) (see also^3^,^18^,^17^); (2) a semi-oval notch for the insertion of the anteromedial branch of the ectopterygoid on the maxilla; and (3) a small fossa corresponding to the impression of the pterygoid at the base of the posterior crest of the quadrate bone. The roughly estimated age of the specimen is around 10 years old, which could match with both *Celestus* and *Diploglossus* as they present similar longevity^29^, but the morphological arguments allow us to identify this fossil as a member of the *Diploglossus* genus.

Among this last genus, the presence of large posterior amblyodont teeth is a character only shared with the Lesser Antillean *D. montisserrati* (see Figs 2B and 3B). In addition, the size of the fossils from Saint-Rose la Ramée (200 mm SVL) is very similar to *D. montisserrati*, since the only two measured specimens were around 180 mm SVL^4^,^30^. Even so, the fossils from Guadeloupe also present a set of characters that do not occur on the *D. montisserrati* specimen observed by us: (1) a medial spine on the supradental shelf of the maxilla delimiting the contact area between the anteromedial branch of the ectopterygoid and the maxilla; (2) two infraorbital foramina on the maxilla instead of three on *D. montisserrati*; (3) a deep coronoid imprint which surpasses the posterior limit of the last dental position in lateral view whereas this imprint is shallower and more posteriorly restricted in *D. montisserrati* and in all the other observed diploglossine lizard skeletons; (4) a well-developed surangular spine that seems shorter in *D. montisserrati*; (5) a notch on the ventral margin of the cotyle of the first sacral vertebra, and two subcentral grooves on the second sacral vertebra. In addition the ratio between the length of the centra of the sacral vertebrae and the maximal width of the vertebrae (comprising the transverse processes) is lower in the fossil (0.34) than for all the observed Diploglossinae, showing that the transverse processes are particularly long compared to the length of the vertebrae in the fossil specimen. Similar results were found in *D. monotropis* (0.39), and to a lesser extent in *D. montiserrati* (0.42). This ratio was higher in all the other observed taxa (*C. costatus* [0.46], *D. millepunctatus* [0.46] and *D. pleii* [0.51]). Yet, because of the low number of observed specimens, we are unable to clearly affirm that all these observed characteristics are not only due to ontogenetic or intraspecific variability, thus we cannot use them to separate the fossil from *D. montisserrati*.

Consequently, this comprehensive set of morphological arguments allows us to identify these fossils as a member of the *Diploglossus* genus, presenting more morphological affinities with *D. montisserrati* than with any other observed Diploglossinae. This is predictable as *D. montisserrati* is the only other known Anguidae in the Lesser Antilles and occurs on Montserrat Island, which is located 60 km north of Guadeloupe. However the fossil also presents some morphological differences with this modern taxon, but we are currently unable to clearly establish if these characters can be interpreted as the skeletal morphological variability of *D. montisserrati*, and more generally of diploglossine anguid lizards, which has not yet been described. Therefore, despite the observed anatomical differences, we are not naming this taxon and we refer to it as *Diploglossus* sp.

## Discussion

The presence of a member of the *Diploglossus* genus in Guadeloupe raises the number of Lesser Antillean Islands presenting evidence of present or past occurrences of *Diploglossus* to three (Montserrat, Basse-Terre and Grande-Terre). On account of their morphological similarity, the Guadeloupe Island and Montserrat galliwasps may derive from the same colonization event by over-water dispersal, which would be younger than the beginning of the geological formation of Basse-Terre 2.79 My ago^31^. However, our study cannot provide new arguments regarding the colonization scenario of *Diploglossus* in the West Indies, mainly because we only observed a single specimen of extant *Diploglossus* from the Greater Antilles (*D. pleii*) and did not find any characteristic morphological traits for this taxon. Still, we should add that it is unlikely that the colonization of the Lesser Antilles by *Diploglossus* was part of the primary colonization event of the Greater Antilles, as no geological data have ever shown the existence of islands as old as 20 Mya in this region.

Past occurrences of anguid lizards in the Lesser Antilles were formerly mentioned by European chroniclers who provided the first descriptions of West Indian faunas during the 17^th^ century. Charles de Rochefort^32^ wrote about a lizard he called a “Brochet de Terre” (land pike) which he drew and described in the following terms: “There are still land pikes in several of these islands (West Indies), that have the same skin and general appearance as our river pikes. However, instead of fins they have four feet that are so weak that the animal has to crawl on the ground, like grass-snakes or, to stick to our comparison, like pikes out of water. The largest cannot exceed fifteen inches long. Their skin is covered with extremely glossy small silver grey scales.” (Rochefort^32^ p. 133–134, translated from French). This description undoubtedly refers to a large skink-like lizard and the anguids are the only West Indian lizards matching this description. The same author also provided the description of another animal that could correspond to an anguid: “By digging in swampy places to build wells or water reservoirs, a very hideous kind of lizard is often found. They are about six inches long. The skin on their backs is black and dotted with small glossy grey scales. The scales of the venter are the same but the skin covering them is yellow. Their heads are small and pointed. They have large mouths armed with many sharp teeth. They have two small eyes but cannot withstand daylight, since right after we extracted them from the ground they tried to dig a hole with their paws […], just as moles do to go wherever they want to. […] Their bite is as venomous as that of the most dangerous snake.” (Rochefort^32^ p. 134, translated from French). The burrowing behavior and the occurrence of the lizard described here in swampy environments could also correspond to an anguid, but the much smaller size than the land pike may point to a different taxon. Yet, the lack of drawings of this animal (the only non-illustrated lizard in the book) and the description of a venomous bite, which is very improbable for a West Indian lizard, could also suggest that the author never personally saw this animal, which could be a juvenile land pike or may never have existed at all. Unfortunately, Rochefort, who visited many West Indian islands, from the southernmost Lesser Antillean Islands to Sainte-Croix in the Virgin Islands, never provides information as regards the location of his naturalist observations. It is thus impossible to establish whether these descriptions refer to the already known Montserrat Galliwasp and Greater Antillean anguids, or to a now extinct species. Jean-Baptiste Du Tertre^33^ also gave a description of a lizard with reduced limbs in the French Lesser Antilles but this animal could either be an anguid or a scincid lizard, which were both well distributed in the area^34^,^35^,^36^. Thus the fossil remains from Saint-Rose la Ramée, Cathédrale de Basse-Terre and Grotte des Bambous are the first tangible evidence of the occurrence of an Anguidae in the Lesser Antilles outside the island of Montserrat.

**Figure 1:**
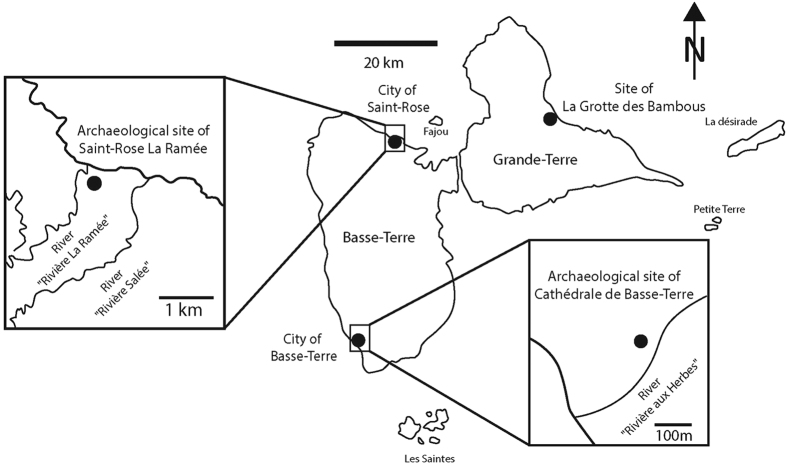
Map of the Guadeloupe Islands and location of the archaeological and natural sites of Saint-Rose la Ramée, Cathédrale de Basse-Terre and Grotte des Bambous on Basse-Terre and Grande-Terre Islands.

**Figure 2:**
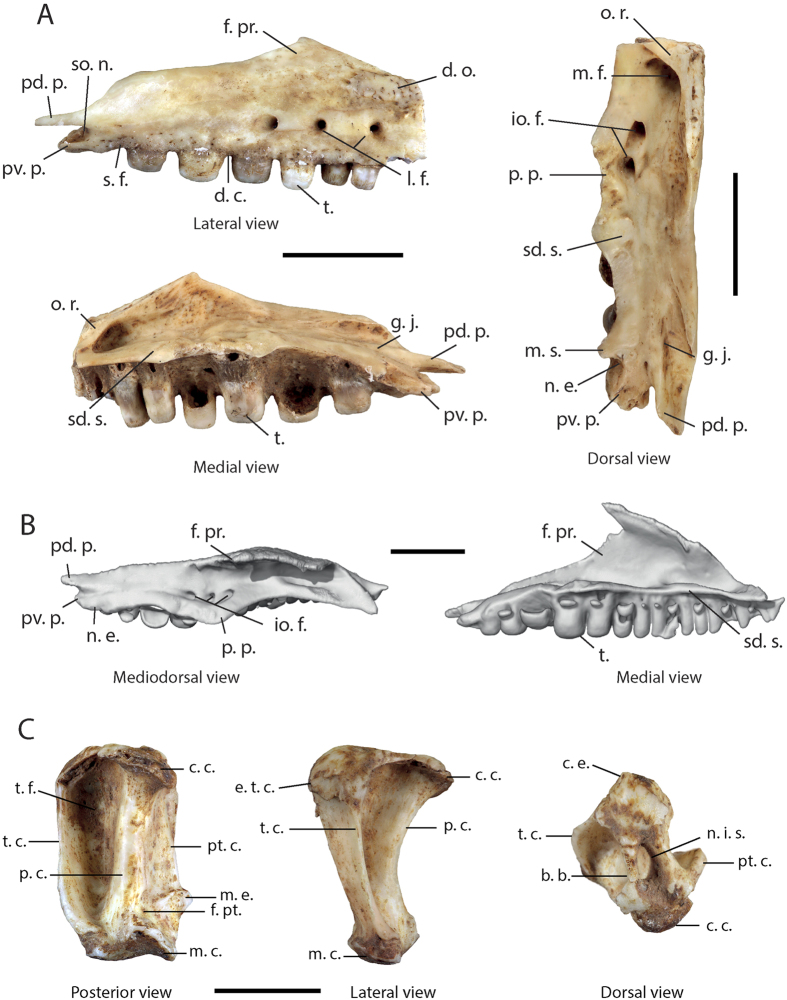
(**A**) Right maxilla of *Diploglossus* sp. from Saint-Rose la Ramée; (**B**) Left maxilla of *Diploglossus montisserrati* (MCZ R-76924); Left quadrate of *Diploglossus* sp. from Saint-Rose la Ramée. Abbreviations: **b. b.**, bony bridge; **c. c.**, cephalic condyle; **c. e.**, columnar epiphysis; **d. c.**, dental crest; **d. o.**, dermal ornamentation; **f. pr.**, facial process; **f. pt.**, fossa for the posterior process of the pterygoid; **g. j.**, groove for the insertion of the anterior ramus of the jugal bone; **io. f.**, infraorbital foramina; **l. f.**, labial foramina; **m. c.**, mandibular condyle; **m. e.**, medial expansion of the pterygoid crest; **m. f.**, maxillary fossa; **m. s.**, medial spine; **n. e.**, notch for the insertion of the anteromedial branch of the ectopterygoid; **n. i. s.**, notch for the insertion of the squamosal; **o. r.**, oblique ridge; **p. c.**, posterior crest; **p. p.**, palatal process; **pd. p.**, posterodorsal process; **pt. c.**, pterygoid crest; **pv. p.**, posteroventral process; **s. f.**, small foramina; **sd. s.**, supradental shelf; **so. n.**, semi-oval notch; **t.**, teeth; **t. c.**, tympanic crest; **t. f.**, tympanic fossa. Scale bar = 5 mm.

**Figure 3:**
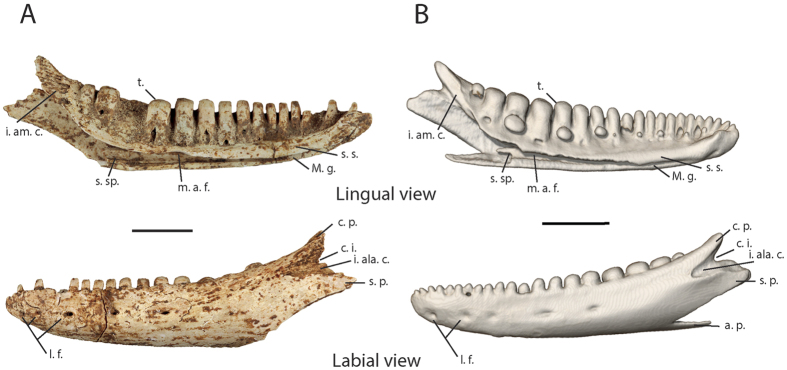
(**A**) left dentary of *Diploglossus* sp. from Saint-Rose la Ramée; (**B**) left dentary of *D. montisserrati*. Abbreviations: **a. p.**, angular process; **c. i.**, coronoid incision; **c. p.**, coronoid process of the dentary; **i. ala. c.**, impression of the anterolateral branch of the coronoid; **i. am. c.**, impression of the anteromedial branch of the coronoid; **m. a. f.**, (dorsal) margin of the anterior alveolar foramen; **M. g.**, Meckelian groove; **l. f.**, lateral foramina; **s. p.**, supra-angular process; **s. s.**, subdental shelf; **s. sp.**, surangular spine; **t.**, teeth. Scale bar = 5 mm.

**Figure 4:**
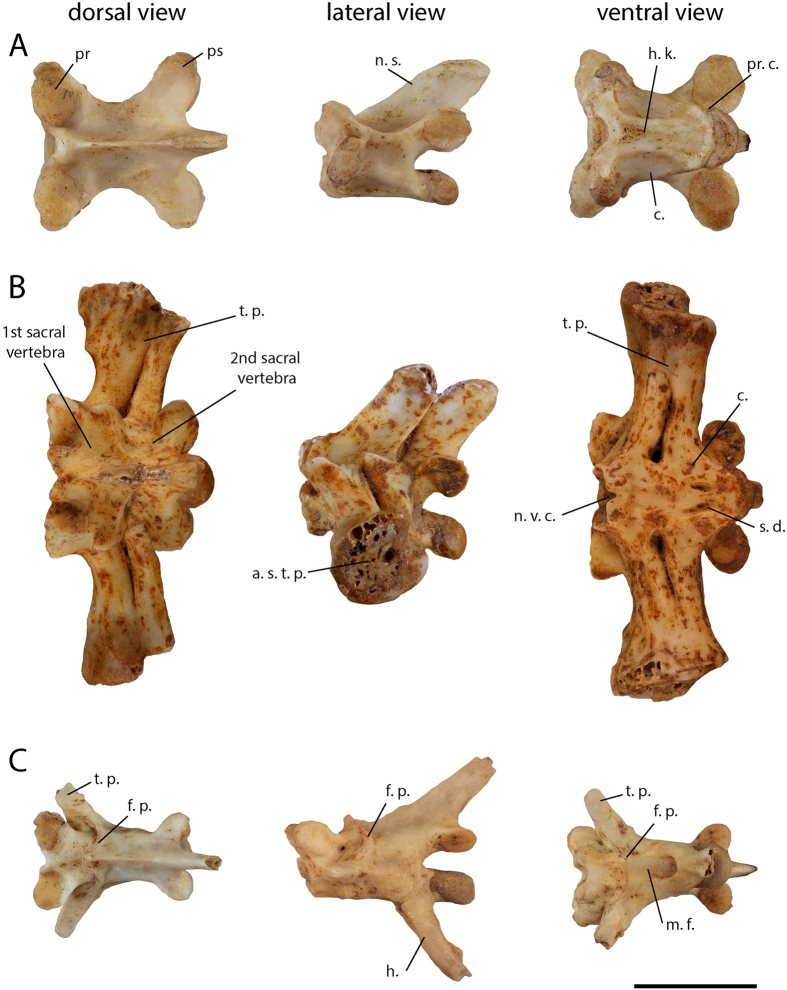
(**A**) Trunk; (**B**) sacral; and (**C**) caudal vertebrae of *Diploglossus* sp. from Saint-Rose la Ramée. Abbreviations: **a.s. t. p.**, articular surface of the transverse processes (sacral); **c.**, centrum; **f. p.**, fracture plane; **h.**, hemapophysis; **h. k.**, hemal keel; **m. f.**, medial furrow; **n. s.**, neural spine; **n. v. c.**, notch on the ventral margin of the cotyle; **pr. c.**, precondylar constriction; **pr.**, prezygapophysis; **ps.**, postzygapophysis; **s. d.**, subcentral depressions; **t. p.**, transverse process. Scale bar = 5 mm.

**Figure 5:**
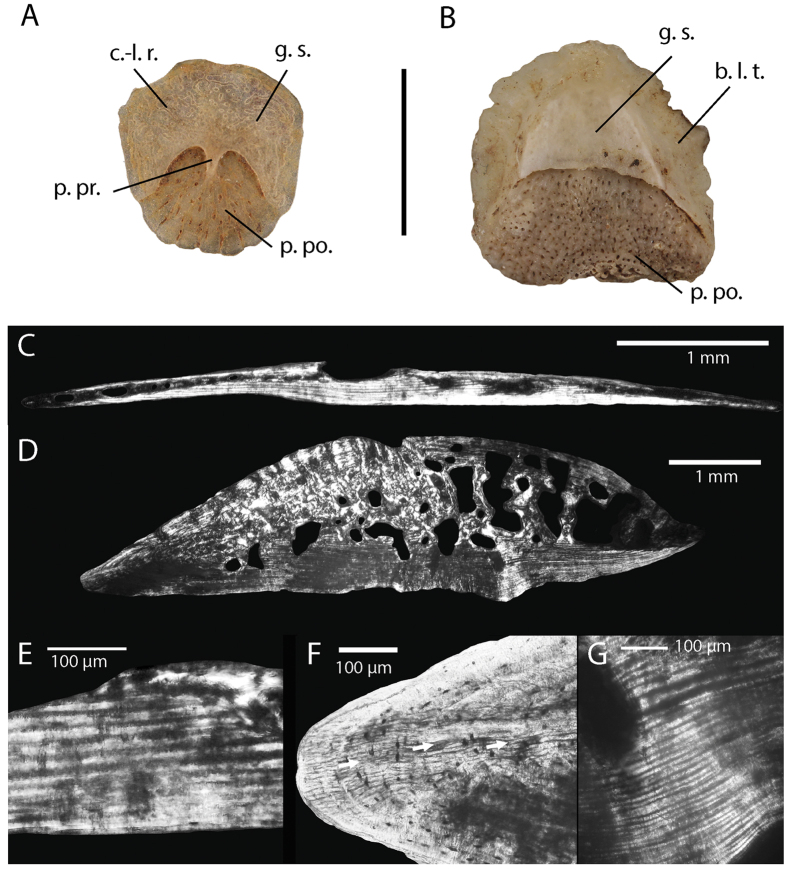
Osteoderms of the (**A**) first morphotype; and (**B**) second morphotype of *Diploglossus* sp. from Saint-Rose la Ramée (superficial view); Cross-section of osteoderms of the (**C**) first morphotype; and (**D**) second morphotype. Detailed view showing the number of Cyclical Growth Marks for each morphotype; (**E**) first morphotype and (**G**) second morphotype) and the occurrence of an additional cortex very rich in Shapey’s fiber transversely oriented in the second morphotype; (**F**) Abbreviations: **c.-l. r.**, cloud-like radix system; **g. s.**, gliding surface; **b. l. t.**, beveled lateral tips; **p. po.**, posterior portion; **p. pr.**, posterior projection.

However, the fact that no fossil *Diploglossus* had ever been mentioned in the Guadeloupe Islands up until now, despite dozens of excavated archaeological and natural deposits^37^,^38^,^39^,^40^ also raises the question of the scarcity of fossil remains. The size of the remains could be an important argument since small animals are less often hunted by human populations and are more difficult to recover during archaeological excavations. However, this does not explain the absence of anguid remains in the natural deposits investigated by Pregill *et al*.^37^ (Pointe du Capucin and Pointe du Vent), and in more recently excavated caves^40^, from which we also studied the osteological material. The behavior of *D. montisserrati* could provide an explanation, since this lizard is strictly nocturnal and is generally found underground or in ground litter. As a consequence, it has only been observed twelve times since its first description by Underwood in 1964^30^, and is probably a very difficult prey to catch, even for nocturnal raptors. Another explanation could be found in the diet of the Montserrat galliwasp, which was, as suggested by its amblyodont teeth^41^,^42^,^43^, reported to feed at least occasionally on crustaceans and mollusks collected in the vicinity of freshwater^4^,^11^. We noted that *Diploglossus* sp. from Basse-Terre and Grande-Terre presents the same tooth morphology as *D. montisserrati*, and as a consequence their feeding behaviors, as well as their environmental niches, may have been comparable. Thus the distribution of *Diploglossus* sp. may also have been restricted to freshwater and wet environments. This could explain its absence in the Grande-Terre natural deposits excavated by Pregill^37^, since this island is nearly flat and much dryer than Basse-Terre^44^. However, the occurrence of a very similar *Diploglossus* dentary to the one described at the Saint-Rose la Ramée site in the site of la Grotte des Bambous on Grande-Terre, in a dryer environment, could refute this hypothesis. Yet this bone presents digestion traces showing that it was carried to the site by a predator, maybe a raptor. Thus, the precise geographic origin of the specimen is difficult to assess. This bias does not concern the specimens from Saint-Rose and Cathédrale de Basse-Terre, which do not bear any traces of avian predators. Moreover, due to the specific geographical situation of these sites near rivers (Fig. 1), these areas were probably suitably humid places for our fossil *Diploglossus*. It is difficult to assess whether the specimens collected from these two archaeological sites were hunted and consumed by human populations, or whether they came to the site of their own accord to feed from the gardens or leftovers, as the remains do not present any traces of cooking or consumption. If the distribution of *Diploglossus* was already restricted to specific areas during pre-Columbian times, this could have made this animal more vulnerable to extinction than other squamates, which are, unlike *Diploglossus*, still extant in Guadeloupe. Another explanation could be that the snails this galliwasp fed on were strongly impacted by the deterioration of the quality of the soil and freshwater due to agricultural and industrial activities since the colonial period, as suggested for other Guadeloupian squamates by Boudadi-Maligne *et al*.^45^. A combination of these two factors, limited geographical distribution and human-induced environmental changes are also evoked by Lenoble *et al*.^46^ to explain the recent extirpation of the terrestrial mollusk *Amphibulima patula* from several Guadeloupe islands, and could also have influenced the extirpation of galliwasps. The role of exogenous predators (mongoose, cat, dogs, rat…), which are often evoked as one of the main factors of the extinction of insular squamates in the Caribbean^47^,^48^ could also be considered as a key factor.

Underwood^4^ suggested that the limited distribution area of *D. montisserrati* and the lack of anguid lizards in the Lesser Antilles could reflect climate change and indicate that *D. montisserrati* was a relictual taxon from a pluvial period. Indeed, paleoclimatic inferences in the West Indies^49^ show that the climate was wetter between 7000 and 3200 BP than it is nowadays, but only the enhanced knowledge of the Lesser Antillean fossil squamate will enable us to test Underwood’s hypothesis. The precise extinction date of *Diploglossus* in Guadeloupe cannot be clearly established due to the absence of a clear chronological attribution of the bone from the “Grotte des Bambous”, but it probably occurred during modern times on account of its association with recently introduced species in the deposits (e.g. mongoose). This hypothesis is reinforced by the fact that no squamate extinction has ever been recorded during Pre-Columbian times in Guadeloupe^34^,^38^,^45^,^50^. However, this animal is so inconspicuous that the possibility that it is still extant in Guadeloupe and has gone unnoticed up until now cannot be definitively ruled out.

These results demonstrate the importance of fossil remains from archaeological and paleontological deposits for investigating past biodiversity and the impact of anthropogenic phenomena. This paper shows that *Diploglossus* should be added to the list of lizard taxa that became extinct over the past centuries in Basse-Terre and Grande-Terre including (*Ameiva*, *Mabuya* and *Leiocephalus*)^35^,^37^. However, further investigations of the Montserrat galliwasp and putative fossils representative of the *Diploglossus* genus in the Lesser Antilles are required to clarify the taxonomic status of the specimens found in Guadeloupe. Indeed, the lack of comparison points and the limits of the use of qualitative skeletal characters to separate closely related species prevent us from specifically identifying these fossils. Future discoveries of fossil *Diploglossus* from the Lesser Antilles, combined with the use of other investigation methodologies, like ancient DNA, will lead to a better understanding of the past history of these lizards.

## Additional Information

**How to cite this article**: Bochaton, C. *et al*. A fossil *Diploglossus* (Squamata, Anguidae) lizard from Basse-Terre and Grande-Terre Islands (Guadeloupe, French West Indies). *Sci. Rep.* **6**, 28475; doi: 10.1038/srep28475 (2016).
